# Quantitative assessment of the financial hardship in the euro area countries

**DOI:** 10.1371/journal.pone.0294886

**Published:** 2024-04-18

**Authors:** Romualdas Ginevičius, Birutė Teodora Visokavičienė, Yuriy Bilan, Marek Lisiński

**Affiliations:** 1 Faculty of Engineering Management, Bialystok University of Technology, Bialystok, Poland; 2 Business Innovation School, Kazimieras Simonavicius University, Vilnius, Lithuania; 3 Faculty of Economics and Management, Czech University of Life Science, Prague, Czech Republic; 4 WSB University, Dąbrowa Górnicza, Poland; Universita degli Studi del Molise, ITALY

## Abstract

The article examines financial hardship (FH) that appears as one of the essential socio-economic-financial categories reflecting a financial burden on society and therefore having a significant impact on the social and economic development of the country. The purpose of this article is to propose and approve a methodology for the complex quantitative assessment of financial difficulty, which allows comparing countries one another. The novelty of the conducted research is manifested by the formed financial hardship adequately exposing a system of indicators and suggesting the transformation of incomparable indicators into the comparable ones. The paper proposes a methodology for the integrated assessment of financial hardship based on multi-criteria methods, which contributes to solving the problems of the social sustainability and economic development of the countries employing different research methods. The proposed methodology provides a possibility of moving to a higher level of research comparing the countries as a whole, in line to the current status of FH. The actual benefits of the carried out research arise from the opportunity to envisage targeted measures for increasing social sustainability subject to the specific situation of the financial hardship of the countries thus removing the burdens of further economic development.

## 1. Introduction

Regardless of the national achievement level, the goal of the socio-economic development of a country is the well-being of people. On the other hand, despite efforts being made, we are witnessing the increasing differentiation of welfare both within and between the countries. The income of part of societies inside the countries is growing disproportionately, whereas the major part is experiencing a growth in material deprivation and fails in meeting the basic needs of personal being. The above introduced negative processes are also driven by the emerging trends in the development of global economy. The wider application of high technology increases production productivity, which changes the structure of qualification consequences on the labour market. The declining birth rate and aging societies are exacerbating this situation. Growing social tensions are accompanied by a rise in crime, the intensification of migration processes, the deterioration of housing or other types of social status, etc.

All above mentioned as well as other present-day-related negative aspects of human existence can be summarized by the concept of financial hardship [1–4]. This is an economic-social category accurately reflecting the well-being of the country’s society, because the welfare state seeks to protect state residents from market forces by ensuring a defined minimum standard of living for all members of society, and particularly for those who, for various reasons (lack of qualifications, fluctuations in labour demand or due to personal qualities), have become ‘participants’ in the labour market. The welfare state becomes an automatic stabilizer of the introduced negative processes and an instrument for managing crises in times of emergencies, i.e. turns into public entities that constantly and directly respond to the resulting consequences of unemployment, loss or depreciation of income, debt burdens, etc. [5–8]. Large-scale financial hardship hinder the social and economic development of the state and distort the relationship between fundamental economic, social and environmental components thus increasing social tensions within the country [1]. FH effects may occur in times of a crisis, as is sometimes asserted today, at the post-crisis stage and even during the periods of rapid economic growth. Thus, it is a constant rather than an episodic phenomenon affecting the economic development situation of the countries [2, 3, 9].

The socio-economic development of the EU Member States (hereinafter the States), especially that of the developing countries, also depends to a large extent on the EU-provided financial support. In order for assistance to be targeted, it is important to determine the actual level of financial hardship faced by the residents of these countries. The established objective is complex due to the fact that financial hardship is by nature an integrated phenomenon that can only be adequately reflected by a system of the indicators. An individual indicator assesses only the aspect taken separately, and therefore fails to describe financial hardship as a whole.

The situation is complicated by the fact that FH indicators have numerous dimensions: they change in opposite trends, i.e. an increase in some values of the indicators improves while a rise in the others–deteriorates the situation and vice versa. The indicators are unequally important in terms of financial hardship. In addition, due to the varying levels of the achieved social and economic development, the peculiarities of the implemented social policy, the use of various social models, etc. the states set different baseline values for these indicators and make them hardly comparable. For example, almost every EU Member State defines a different size of minimum income that guarantees the necessary standard of living; owing to the diverse ways and levels of establishing labour relations, the states determine the at-risk-of-poverty threshold etc. employing different methods. As a result, it is tricky to adequately assess the social sustainability and well-being of the country’s society, and thus financial hardship as a whole. At present, the EU lacks a universally accepted methodology that allows the integrated assessment of financial hardship at the national level, which poses difficulties in comparing the states with each other. As mentioned above, this reduces the effectiveness of EU financial assistance provided to the states and prevents them from developing a targeted FH reduction strategy. The latter requires fundamental changes in the management of national economic resources in connection with the transformation of the management institutions of the financial sector, including public finances and other social, monetary and fiscal policies, etc. [9].

The objectives of this study cover proposals for a methodology for the integrated quantitative assessment of financial hardship granting a comparison of the states with each other and ranking the EU Member States in line to the achieved level of financial hardship thus classifying these states in consonance to the evident limits of changes in financial hardship, which facilitates creating conditions for targeted strategies to reduce financial.

The purpose of the article is to propose and approve a methodology for the complex quantitative assessment of financial difficulty, which allows comparing countries one another.

The following tasks are set for this study: based on the proposed methodology, to rank the countries of the EU euro zone according to the achieved FS level; to group these countries according to the emerging limits of the change in financial difficulty, which would enable the formation of purposeful strategies for its reduction.

## 2. Literature review

The phenomenon of financial hardship has been investigated for decades. The issue is addressed in various aspects. Two research-related trends cover the impact of changes in individual socio-economic factors on the social sustainability of human capital and the integrated assessment of financial hardship as a multidimensional phenomenon.

A study of the impact of a national social policy on financial hardship documented that the policy primarily affected fundamental social evils such as human unemployment and poverty [10]. Much attention is paid to the financial sector the effectiveness and governance of which have been found to be decreasing in recent years. The research conducted by the International Monetary Fund shows this largely contributes to a growth in the economic inequality and exclusion of people [9, 11, 12]. Moreover, the examined impact of financial hardship on a country’s gross domestic product per capita disclosed a very strong correlation (*r* = 0.77) [4]. Another aspect of the carried out research involved the impact of technology and robotics on the scale of the labour market, which resulted in the emergence of new problems [13]. Financial hardship is also under the impact of the national monetary policy that must ensure a balance between money supply and demand. This is especially true in crisis and post-crisis years. Despite the significance of investigation in the first trend, these periods do not allow for the integrated assessment of financial hardship that is of multidimensional nature [14].

For this reason, most research is specifically focused on finding an integrated indicator for financial hardship. Different approaches to the social sustainability indicator for human capital are adopted. Some authors considered it was poverty, because this category combined the essential manifestations of the above mentioned sustainability [15]. Yet, human income is the key measure for reflecting poverty [16]. Other authors argued that the discussed sustainability was not reflected in poverty but in economic vulnerability [17]. However, this approach was criticized due to the fact it was difficult to define this particular concept of financial hardship. Vulnerability mainly means the risk, i.e. likelihood of experiencing financial hardship. Likelihood is difficult to measure [14], and hence it follows that the concept of poverty is more closely linked to financial hardship than to income.

Thus, an attempt to reflect financial hardship applying one specific indicator is made. Even though such indicators are integrated, i.e. combine a number of important manifestations of the examined phenomenon (in our case, financial hardship), still, this is a limited approach, because it inadequately assesses all major aspects of financial hardship exhibited in reality. With this in mind, the concepts integrally assessing financial hardship have been developed. The most prominent concept is the latent class model of financial hardship based on three indicators from the European Union Statistics on Income and Living Conditions (EU-SILC) dataset [18], that is, equivalized disposable income, the ability to make ends meet, i.e. consolidate financial statements, and material deprivation, all measured at the household level [19].

Model allows determining the status of the three main components of financial hardship and thus dividing the population of the State into two classes–people experiencing (Class 1) and those not subjected (Class 2) to financial hardship. The evaluation of the suggested model and other similar proposals [20] may lead to the following considerations. First, a question on the integrated model arises, as this allows the States to be compared on the basis of the individual components of financial hardship only. Thus, it is hardly possible to compare the States on the basis of a general indicator for financial hardship, because it is unclear how to measure the level of social exclusion in the State applying a single general indicator bearing in mind this is an important aspect of financial hardship. Literature sources point to the multidimensional nature of the indicators reflecting FH as the main obstacle to quantifying financial hardship. The available methodologies address this problem by coding indicator values into categories, which is a greatly simplified approach ‘pushing’ reality into a narrow-range scoring system. Attributing the values of income-related indicators, i.e. poverty, to certain categories is highly questionable. The obtained values are incomparable between the States, as each one determines a different at-risk-of-poverty threshold.

To sum up the current situation of the quantified FH status, a methodology that allows including the indicators in the model regardless of indicator dimension is required. The values of the indicators should be transformed into the comparable ones to combine all indicators into a single aggregate conforming to their importance, which may create conditions for making comparisons between the States. This may assist in the social development of the research base throughout the States.

## 3. Research methodology

### 3.1 Building a system of the indicators for determining financial hardship at the national level

The analysis of financial hardship, as a phenomenon, throughout the EU Member States allows identifying the main aspects of the occurrence covering material deprivation, poverty and income. Considering the format followed, all aspects are integrated, i.e. belong to the dimensions the value of which cannot be determined directly. In this case, partial indicators reflecting values are appropriately combined into a single aggregate [21]. In turn, the combination of the latter results in an index of the investigated phenomenon, which is national FH.

The analysis of literature sources demonstrates that material deprivation is sufficiently adequately reflected by two indicators–material deprivation and severe material deprivation, poverty is manifested by the at-risk-of-poverty rates of working and non-working age population and income is exposed by income size and inequality. Thus, the following set of FH indicators for the States emerges:

1. material deprivation [22] is an indicator for the inability to afford things that most people consider desirable or even necessary to live a decent life. In line to the EU-SILC methodology [23], an indicator approved by the Social Protection Committee calculates the percentage of the population that fail to afford at least three of the nine items [22].
2. severe material deprivation, percentage of the population that fail to afford at least four of the nine items [22];
3. at-risk-of-poverty rate of 60% of persons under 18 years of age, the indicator is defined as the share of persons of a defined age with an equivalised disposable income below the risk-of-poverty threshold, which is set at 60% of the national median equivalised disposable income (after social transfers) [24].
4. at-risk-of-poverty rate of 60% of persons under 65 years of age, the indicator is defined as the share of persons of a defined age with an equivalised disposable income below the risk-of-poverty threshold, which is set at 60% of the national median equivalised disposable income (after social transfers) [24].
5. at-risk-of-poverty rate of persons aged 24–64 [24].
6. disposable income per capita defined as total income conforming to the composition of GDP (wages-taxes, property-taxes, dividends, rent, bond interest, shares, etc.) and other types of income (pension, scholarship) [23].
7. the income inequality indicator is defined as the ratio of the total income received by 20% of the highest-income population (highest quintile) to the income earned by 20% of the lowest-income population (lowest quintile).
8. *The Gini coefficient* of equivalised *disposable income* is a measure aimed at highlighting the difference between the distribution of equivalent disposable income following social transfers and completely equal distribution [25].

FH indicators differ from each other in the units of measurement, a varying format and an impact on financial hardship and comparability, i.e. some indicators are not comparable between the States due to different calculation methodologies. In order to combine the indicators into a single aggregate integrally reflecting the current state of national FH, they primarily need to be appropriately transformed to become fully comparable.

It has been found that the following indicators are currently comparable: material deprivation; severe material deprivation; disposable income per capita of the country; income inequality and the Gini coefficient; incomparable − 60% of people under 18 are at risk of poverty; at-risk-of-poverty rate 60% of people over 65 and those aged 24–64 at-risk-of-poverty.

Transforming the values of incomparable indicators into the comparable ones is required thus equalizing trends in the varying indicators, making all indicators dimensionless and determining the significance of the impact of the indicators on financial hardship. This will allow the indicators to be combined into a single aggregate in an appropriate way.

There is a need to compare the values of incomparable (unique) indicators with comparable ones, thus smoothing the trends of changing indicators, turning all indicators dimensionless and determining the significance of the impact of indicators on financial hardship. This will allow the indicators to be properly aggregated into a single summary.

### 3.2 Multi-criteria evaluation of the financial hardship of the EU Member States

The quantification of the FH status of the EU Member States starts with the transformation of indicator values into the comparable ones. The values cannot be compared for two reasons: first, the States estimate different baseline poverty rates; second, the indicators change in opposite trends, i.e. some show an maximizing while others a minimizing trend and are expressed in different dimensions.

#### Transforming the values of poverty indicators by making them comparable between the States

Due to different at-risk-of-poverty thresholds determined by the States, indicators *Q*_3_, *Q*_4_ and *Q*_5_ are not comparable. For example, in 2018, per capita income corresponding to the at-risk-of-poverty threshold was € 7,717 in Slovenia and € 13,067 –in Belgium. Thus, a Belgian citizen with per capita income 1.7 times higher than that of a Slovenian citizen is at risk-of-poverty. The parties will be comparable in the case of the following recalculation:

$${\tilde{Q}}_{3j}=\frac{Q_{3j}}{Q_{3j}^{\text{min}}};$$ (1)

$${\tilde{Q}}_{4j}=\frac{Q_{4j}}{Q_{4j}^{\text{min}}},$$ (2)

where ${\tilde{Q}}_{3j}$ and ${\tilde{Q}}_{4j}$–the at-risk-of-poverty rate indicators re-calculated in the *j*-th country; $Q_{3j}$ and $Q_{4j}$–per capita income set in the *j*-th country; lower per capita income falls into the category of people at risk-of-poverty; $Q_{3j}^{\text{min}}$ ir $Q_{4j}^{\text{min}}$–per capita income lower which a person falls into the category of people at risk-of-poverty in the country having the smallest income.

To make indicator *Q*_5_ comparable, the following transformation of the indicator value is necessary:

$${\tilde{Q}}_{5j}=\frac{S^{\text{min}}N_{j}}{S_{j}},$$ (3)

where ${\tilde{Q}}_{5j}$–the re-estimated at-risk-of-poverty rate of the *j*-th country; *N*_*j*_–the percentage of the working population aged 24–64 falling into the category of people at risk-of-poverty vs the total population of the country; *S*_*j*_–the estimated at-risk-of-poverty rate of the working population in the *j*-th country, euros per year; *S*^min^–the estimated at-risk-of-poverty rate of the working population in the *j*-th country having the lowest rate.

#### The equalization of trends in the varying values of the indicators

To equalize trends in the varying indicators, all indicators must be transformed into either maximizing or minimizing ones. The maximizing values of the indicators are calculated in the following way [21]:

$${\hat{q}}_{ij}=\frac{q_{ij}}{q_{ij}^{\text{max}}},$$ (4)

where ${\hat{q}}_{ij}$ –the maximizing value of the *i*-th indicator in the *j*-th country; *q*_*ij*_–the value of the *i*-th indicator in the *j*-th country; $q_{ij}^{\text{max}}$–the highest value of the *i*-th indicator in the *j*-th country.

The values of the indicators are maximized as follows:

$${\hat{\bar{q}}}_{ij}=\frac{q_{ij}^{\text{min}}}{q_{ij}},$$ (5)

where ${\hat{\bar{q}}}_{ij}$ –the minimizing value of the *i*-th indicator in the *j*-th country; $q_{ij}^{\text{min}}$–the lowest value of the *i*-th indicator in the *j*-th country.

#### The conversion of indicator values to dimensionless

The conversion of indicator values into dimensionless is carried out applying the normalization procedure. The order of normalization is subject to the method of multi-criteria evaluation. The most widely used SAW method provides for the below given normalization technique [21]:

$${\tilde{q}}_{ij}=\frac{q_{ij}}{\sum_{i=1}^{n}q_{ij}},$$ (6)

where ${\tilde{q}}_{ij}$–the normalized value of the *i*-th indicator in the *j*-th country; *n*–the number of the indicators $\left(i=\bar{1,n}\right)$.

The multi-criteria TOPSIS method provides data normalization as follows [21, 26, 27]:

$${\tilde{q}}_{ij}=\frac{q_{ij}}{\sqrt{\sum_{i=1}^{n}q_{ij}^{2}}}.$$ (7)

The multi-criteria VICOR method provides data normalization as follows [27]:

$${\tilde{q}}_{ij}=\frac{\frac{\text{max}\;q_{ij}-q_{ij}}{j}}{\frac{\text{max}\;q_{ij}}{j}-\frac{\text{min}\;q_{ij}}{j}}.$$ (8)

#### Determining the weights of the indicators

Various methods for determining indicator weights are known. Some techniques such as the direct assessment of indicator weights, i.e. when experts immediately indicate the importance of the indicators in parts of a unit and clarify the weights of the indicators based on their ranks, etc., are attributed to simple methods [28]. Other techniques are more complex and allow assessing a larger number of the indicators [29, 30].

Determining the weights of the indicators is usually based on an expert survey that is biased in all cases, which imposes certain limitations to selecting an assessment method. On the other hand, in any case, the technique that largely minimizes the impact of the prejudice factor is the most appropriate. Thus, the FARE method was chosen to assess the importance of FH indicators for the EU Member States. At present, the method is gaining wider application [31–34]. The core of the technique is similar to that of the AHP method because the matrix of the interacting indicators is filled in both cases. The interaction is assessed in terms of the impact of the indicators on the investigated phenomenon (IP). In this manner, a square matrix is formed to determine the weights of the indicators following appropriate calculation procedures (Table 1).

**Table 1:** A matrix for assessing the weights of the indicators employing the FARE method. Source: created by the authors.

	Indicators							$\sum_{i=1}^{m}P_{i}$
	1	2	3	…	*i*	…	*m*	
1		*P*_12_	*P*_13_	…	*P*_1*i*_	…	*P*_1*m*_	*P*_1_
2	−*P*_21_		*P*_23_	…	*P*_2*i*_	…	*P*_2*m*_	*P*_2_
3	−*P*_31_	−*P*_32_		…	*P*_3*i*_	…	*P*_3*m*_	*P*_3_
	⋮	⋮	⋮		⋮	⋮	⋮	⋮
*i*	−*P*_*i*1_	−*P*_*i*2_	−*P*_*i*3_	…		…	*P*_*im*_	*P*_*i*_
	⋮	⋮	⋮	⋮	⋮		⋮	⋮
*m*	−*P*_*m*1_	−*P*_*m*2_	−*P*_*m*3_	…	−*P*_*mi*_	…		*P*_*m*_

**Fig 1:**
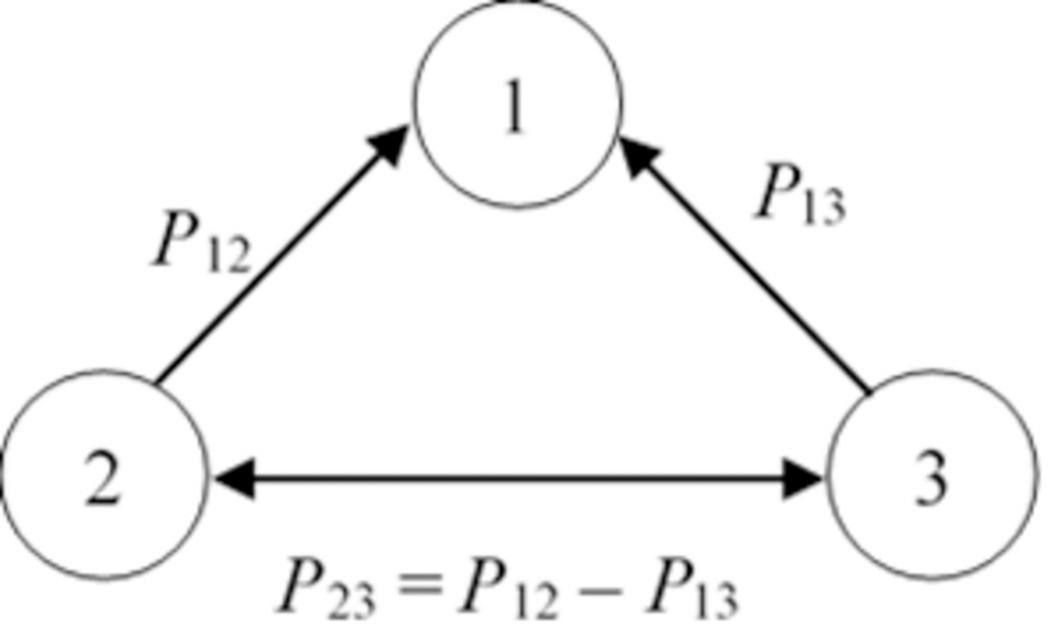
A triangle determining the interaction strength of the indicator. Source: made by the authors.

The FARE method views the indicators reflecting IP as a system, i.e. as a whole of the interacting elements. The major feature of the system is stability ensured by internal balance which requires equalizing two principal parameters for system elements–interaction trend and strength. This circumstance allows developing the interaction system for all indicators solely on the basis of the impact of all indicators on IP arranged in a descending order and strength compared to the impact of the most important indicator determined by experts. The interaction, trend and strength of all other indicators stem from requirements for system equilibrium achieved on account of the 1^st^ line of the matrix made of triangles. The values of two margins are known, and impact trends arise from the previous ranking of their importance to the investigated phenomenon (Fig 1).

The known values of the impact of the 2^nd^ and 3^rd^ indicators on IP are shown in the triangle provided in Fig 1 and compared with the impact of the most important 1^st^ indicator. Next, the value of the impact of the 2^nd^ indicator compared to the 3^rd^ indicator of lower importance is found and is equal to the difference between *P*_12_ and *P*_13_: *P*_23_ = *P*_12_ ‒ *P*_13_. Thus, a comparative impact of all other elements of the matrix on the investigated phenomenon is determined.

In contrast to the AHP method, experts fill in only the 1^st^ line of the matrix, whereas the rest of the lines are filled in by simple calculations. This results in a fully agreed matrix that allows determining the adequate weights of the indicators.

Based on Table 1, the part of the matrix above the diagonal is filled in following the indicated order. The other part is inversely symmetrical to the first one.

The weights of the indicators *ω*_*i*_ are determined by the FARE method as follows:

$$\omega_{i}=\frac{P_{i}^{F}}{P_{S}},$$ (9)

where $P_{i}^{F}$–the actual aggregation of the impact of the *i*-th indicator on the potentials of the investigated phenomena; *P*_*S*_–the potential of the impact of the whole system of the indicators.

Value $P_{i}^{F}$ is determined as follows:

$$P_{i}^{F}=P_{i}+P_{1},$$ (10)

where *P*_*i*_–the aggregated impact of the *i*-th indicator on IP (Table 1); *P*–the basic potential of the impact of the indicators.

Value *P* is equal to

$$P=S\left(m-1\right),$$ (11)

where *S*–assessment scale (10, 100 points, etc., depending on the number of the indicators).

Value *P*_*S*_ is determined in the following way:

$$P_{S}=P∙m.$$ (12)

#### Multi-criteria evaluation of national FH

The practice of the integrated-quantitative assessment of complex phenomena demonstrates that multi-criteria methods are most appropriate [21, 27] and broadly applied. The techniques cover the quantitative assessment of the status of economic and social [35–38] as well as engineering-technological [39–47] processes. This is due to the fact that multi-criteria methods make it possible to combine multidimensional indicators into a single aggregate, vary in opposite trends and are unequally important in terms of the investigated phenomenon.

The philosophy of multi-criteria evaluation is reflected by the SAW (Simple Additive Weighting) method expressed as [21]:

$$K_{j}=\sum_{i=1}^{m}\omega_{i}∙{\tilde{q}}_{ij},$$ (13)

where *K*_*j*_–the value of the multi-criteria evaluation of the status of the investigated phenomenon in the *j*-th country; *ω*_*i*_–the importance of the *i*-th indicator to the status of the investigated phenomenon ($i=\bar{1,m}$, *m*–the number of the indicators); ${\tilde{q}}_{ij}$–the normalized value of the *i*-the indicator in the *j*-th country.

Formula (13) shows that value and importance are the two dimensions that express each indicator in multi-criteria evaluation. The required status for determining the weights of the indicators is

$$\Sigma^{n}\omega_{i}=1,0.$$

## 4. Empirical research

The quantification of national FH is based on the system of the indicators reflecting hardship. The values of the indicators have been obtained from various statistical sources [48–50] [S1 Annex].

Based on formulas (1)‒(3), non-comparable indicators were transformed into the comparable ones. Considering a smaller number of the indicators for maximizing rather than minimizing financial hardship, in consonance to formula (5), they were made the minimizing ones. Following the above two procedures, the normalization of indicator values was performed in keeping with Formula (12), i.e. all indicators were made dimensionless, and thus comparable.

As provided by the FARE method, calculating the importance of FH indicators starts from determining the most important indicator having the greatest impact on financial hardship. For that purpose, expert evaluation was carried out. The experts were the representatives of scientific institutions and the staff directly involved in the analysis of macroeconomic development processes of the EU Member States. The experts ranked all indicators conforming to their importance to national FH. The consistency of expert opinions was checked on the basis of concordance coefficient *W* and Pearson *χ*^2^ [51]. The value of *W* was found to be equal to 0.75. The actual value of Pearson criterion *χ*^2^ was equal to 36.665 and the critical value made 14.067. Thus, expert opinions were agreed to be consistent. The following ranking results of indicator importance were obtained (Table 2).

**Table 2:** The importance ranks and impact of FH indicators for the EU Member State. Source: created by the authors.

Indicator	Material deprivation	Severe material deprivation	At-risk-of-poverty rate of 60% of persons under 18 years of age	At-risk-of-poverty rate of 60% of persons under 65 years of age	At-risk-of-poverty rate of persons aged 24–64	Disposable income per capita	Income inequality	Gini coefficient
Importance rank	2	1	3	4	5	6	7	8
1^st^ line of the matrix (Table 1)	12		28	32	39	50	56	62
Weights of the indicators, *ω*_*i*_	0.158	0.175	0.135	0.129	0.119	0.103	0.095	0.086

Based on the above introduced methodology, the 1^st^ line 1 of the matrix had to be completed next (Table 1). The experts were asked to answer the question: ‘In a 100-point scale, indicate the impact of the listed indicators on national FH compared to the main factor, i.e. severe material deprivation, estimated as having 100-point impact’. The obtained results of the agreed expert opinions were equal to *W* = 0.68, *χ*^2^_*f*_ = 8.42 > *χ*^2^_*kr*_ = 2.571. Consequently, expert opinions were found to be consistent. The calculation results of indicator impact on national FH are given in Table 2.

The matrix in Table 1 shows that the lower is the significance rank of the indicator, the greater loss of aggregated impact *P*_*i*_ on the investigated phenomenon is observed. In this particular case, the matrix provides the following formula:

$${\tilde{P}}_{i}=S-P_{i},$$ (14)

where ${\tilde{P}}_{i}$–the transformed impact of the *i*-th indicator on the investigated phenomenon; *P*_*i*_–the impact of the *i*-th indicator on the investigated phenomenon.

With reference to Formula (14), the 1^st^ line of the matrix takes the following form (Table 2).

On the basis of Fig 1 and Table 1, the matrix assessing indicator weights can be completed employing the FARE-M method as well as determining the weights of the financial hardship indicator (Table 3).

**Table 3:** Multi-criteria evaluation results of financial hardship in the EU Member States in 2018. Source: created by the authors.

No	Member State	*k*_*j*_	No	Member State	*k*_*j*_
1	Belgium	0.045461	11	Lithuania	0.083281
2	Germany	0.039622	12	Luxembourg	0.040679
3	Estonia	0.046793	13	Malta	0.03553
4	Ireland	0.043992	14	Netherlands	0.047231
5	Greece	0.096813	15	Austria	0.03625
6	Spain	0.052885	16	Portugal	0.047642
7	France	0.045349	17	Slovenia	0.04924
8	Italy	0.056993	18	Slovakia	0.062412
9	Cyprus	0.062318	19	Finland	0.03784
10	Latvia	0.063612			

The normalized values and weights (Table 2) of all indicators are known, which allows for the immediate multi-criteria evaluation of the financial hardship of the EU Member States applying Formula (14). Calculation results are given in Table 3.

Table 3 shows that the financial hardship of the individual EU Member States varies widely. For example, the financial hardship of Greece ranked in the last line is 2.7 times greater compared with that of Malta. Calculation results allow for grouping all examined Member States in line to their financial hardship (Table 4).

**Table 4:** Grouping the EU Member States in line to the FH status in 2018. Source: created by the authors.

Limits of variations in the indicator for financial hardship		
< 0.04	0.04‒0.05	0.05>
1. Austria	1. Ireland	1. Greece
2. Malta	2. Belgium	2. Spain
3. Finland	3. Estonia	3. Italy
4. Germany	4. Luxembourg	4. Cyprus
	5. Netherlands	5. Latvia
	6. Portugal	6. Lithuania
	7. France	7. Slovakia
	8. Slovenia	

Table 4 shows that the financial hardship of the 1^st^ group of the EU Member States is on average by more than 1.2 times lower compared to that of the 2^nd^ group and by more than 1.8 times − compared to that of the 3^rd^ group. The financial hardship of the 2^nd^ group of the Member States is on average by almost one and a half times lower than that of the 3^rd^ group of the States.

The quantitative assessment of the FH status of the States for the desired period opens wide possibilities for analysing demographic, cultural, criminogenic and other aspects of social development.

## 5. Discussion

FH indicators by State demonstrate numerical differences and correlations with FH characteristics in each of the States. For example, the financial hardship of the Greek population expressed in the FH index is 3 times higher than that of Malta. Greece has the highest estimated financial hardship and material deprivation amounting to 33.6% of the total population 16.7% of which suffers from the greatest severe material deprivation. (Table 2).

At first glance, the at-risk-of-poverty rate in Greece would appear to be lower than that in Malta, but the at-risk-of-poverty threshold is different and incomparable between the two States. In 2019, the population of Greece was at-risk-of-poverty when disposable income per capita made € 5,052 while that in Malta was € 9,564 [50]. Even greater differences were observed between Luxembourg, where the at-risk-of-poverty threshold rate was set at € 22,321 [50], and Lithuania and Latvia, where the at-risk-of-poverty threshold rate was set at € 4,838 and € 4,734 respectively [50]. Therefore, given the at-risk-of-poverty rate, the financial difficulty indicator can’t comparable in either the euro area or the EU.

There are even more differences between the States that are not assessed using both comparable and non-comparable indicators, for instance, geographical and climatic conditions determining household expenditure. Although the Member States of the eastern Baltic region also face significant financial hardship, however, their housing and energy costs are hardly compared with those of the southern EU region, Greece, Italy, etc. Thus, financial hardship conforming to material deprivation should be methodologically matched.

It is clear that efforts to equalize the system of FH indicators in the EU Member States and to use a scientifically based, standard and comparison-focused methodology between the States would induce improvements to the status determining financial hardship, stimulate appropriate solutions to management problems, prompt the transformation of social policy towards reducing financial hardship and promote the resilience of the population to economic downturns through economic policy measures.

## 5. Conclusions

1. Despite the importance of financial hardship for national economic and social in particular development, the research status of this phenomenon is insufficient. The main shortcomings of such studies are as follows: first, FH indicators have not been fully highlighted and discussed, and therefore a single system for the indicators reflecting financial hardship is not available; second, ranking the EU Member States in line to their financial hardship is impossible, because some of the indicators showing financial hardship are hardly compared between the States; third, no methodology for the integrated assessment of the national FS status has been proposed, which makes it impossible to assess financial hardship as a whole, i.e. express FH in a single index. This prevents the States from being ranked conforming to their financial hardship and from adopting best practices in financial hardship management. For the reasons listed above, the assessment of individual FH aspects is limited to the national rather than international level.
2. FH indicators are not comparable between the States because their values are subject to the different at-risk-of-poverty threshold set in each State. A different percentage of people at the risk-of-poverty is identified by the EU Member States, which is another reason for indicator incomparability. To make the indicators comparable, an appropriate transformation of their values is required.
3. FH indicators at the national level are expressed in different dimensions; trends in the varying indicators do not coincide, i.e. some indicators are maximizing while others are minimizing and the importance of indicator effects on financial hardship is observed. Multi-criteria methods are best suited to combine such controversial indicators into a single aggregate. The methods express each indicator in two quantities–value and importance. Values can be found in international statistical publications and importance is determined by experts. The FARE method used in the article allows estimating the importance of the indicators quite accurately and assesses both the impact on the investigated phenomenon and interaction strength.
4. The integrated assessment of the national FH status opens up a wide range of possibilities for the causal analysis of demographic, cultural, criminogenic and other aspects of social development. In addition, the division of the States into the groups subject to the FH status allows the countries to envisage targeted measures for increasing social sustainability and removing the burdens of subsequent economic development, which is a trend towards further research related to financial hardship.
5. In order to apply successfully the proposed Financial Difficulty Assessment methodology, transformed values of FH indicators, primarily reflecting poverty, should be presented in international databases, so that they can be compared among countries.
6. Further research directions for the quantitative assessment of the state of FH could be as follows: improvement of the system of indicators reflecting financial difficulty both in terms of their number and structure, improvement of methods of combining indicators into the FH index, etc.

## Supporting information

S1 Annex (DOCX)
