# Supplementary material for: Quantitative assessment of the financial hardship in the euro area countries
Source: PLoS One. 2024 Apr 18;19(4):e0294886. doi: 10.1371/journal.pone.0294886 (PMC11025939; doi:10.1371/journal.pone.0294886)
Supplement: S1 Annex — (DOCX) [file pone.0294886.s001.docx]

Annex 1

The data presented in our article "Quantitative Assessment of the Financial Hardship in the Euro Area Countries" are the results of the conducted research, they will be available to other researchers when the article is published. Therefore, we disclose the primary statistical data on the basis of which the study was conducted and their sources in the annex 1.

Table A1. Indicators that affect Financial Hardship

| Euro area  countries  /indicators | Material deprivation^4^,  Percent from total population | Threshold of poverty,  €/per year^2^ | Material deprivation with difficulty,  Percent from total population^5^ | At risk of poverty^2^ (60%)  Age<18/  Age>=65  €/per year | At risk of poverty^2^ of employers  Age 24-64, % from total population | Income inequality^1^ | Disposable income per one inhabitant, €/per year /  Gini^3^ |
| --- | --- | --- | --- | --- | --- | --- | --- |
| BE | 10.7 | 13934 | 5 | 13067/8189 | 5.1 | 3.79 | 28386/0.211 |
| DE | 7.8 | 13308 | 3.1 | 14421/16004 | 9.0 | 5.07 | 26622/0.272 |
| EE | 9.9 | 7023 | 3.8 | 14403/42370 | 9.5 | 5.07 | 13100/0.294 |
| IE | 11.6 | 13762 | 4.9 | 16812/9584 | 4.8 | 4.23 | 32113/0.299 |
| GR | 33.6 | 5052 | 16.7 | 18130/9574 | 10.9 | 5.51 | 10187/0.286 |
| ES | 13.9 | 8685 | 5.4 | 28248/13109 | 13.0 | 6.03 | 20082/0.330 |
| FR | 11.0 | 14210 | 4.7 | 14297/10051 | 7.1 | 4.23 | 26030/0.270 |
| IT | 16.8 | 10088 | 8.5 | 23247/15205 | 12.3 | 6.09 | 21474/0.311 |
| CY | 27.0 | 8955 | 10.2 | 18354/16235 | 7.4 | 4.29 | 19881/0.311 |
| LV | 21.0 | 4734 | 9.5 | 17239/43952 | 8.7 | 6.78 | 9906/0.340 |
| LT | 23.1 | 4838 | 11.1 | 19246/32931 | 8.3 | 7.09 | 9351/0.345 |
| LU | 4.8 | 22321 | 1.3 | 15402/6353 | 13.5 | 5.79 | 53486/0.254 |
| MT | 8.7 | 9564 | 3.0 | 21311/35782 | 6.4 | 4.28 | /0.290 |
| NL | 6.5 | 14717 | 2.4 | 14015/4585 | 6.1 | 4.05 | 29400/0.253 |
| AT | 6.3 | 15503 | 2.8 | 15587/12824 | 8.0 | 4.04 | 30710/0.259 |
| PT | 16.6 | 5997 | 6.0 | 18799/18466 | 9.6 | 5.22 | 12478/0.326 |
| SI | 10.4 | 7903 | 3.7 | 7716/15027 | 6.0 | 3.38 | 15982/0.233 |
| SK | 17.2 | 4876 | 7.0 | 20252/7190 | 6.0 | 3.03 | 8973/0.225 |
| FI | 8.0 | 14912 | 2.8 | 8601/11258 | 2.9 | 3.65 | 29616/0.235 |

^1^ The ratio of total income received by the 20% of the population with the highest income (highest quintile) to the income received by the 20% of the population 20% of the population receiving the least income (lowest quintile). Income must be understood as equivalent income.

<https://ec.europa.eu/eurostat/web/income-and-living-conditions/database>

^2^ At-risk-of-poverty rate by age - The indicator is defined as the share of persons of a defined age with an equivalised disposable income below the **risk-of-poverty threshold**, which is set at 60% of the national median equivalised disposable income (after social transfers).

<https://ec.europa.eu/eurostat/databrowser/view/TESSI014/default/table?lang=en>

^3^ The Gini**of equivalised disposable income** measures the extent to which the distribution of equivalised disposable income after social transfers deviates from a perfectly equal distribution. It is a summary measure of the cumulative share of equivalised income accounted for by the cumulative percentages of the number of individuals. Its value ranges from 0 (complete equality) to 100 (complete inequality).

<https://www.oecd.org/social/income-distribution-database.htm>

^4^Material deprivation 4 includes indicators related to economic burden, durable goods, housing and housing environment.

<https://ec.europa.eu/eurostat/web/income-and-living-conditions/database>

Note

The authors relied on the data available at the time
